# Quadratically convergent algorithm for computing real root of non-linear transcendental equations

**DOI:** 10.1186/s13104-018-4008-z

**Published:** 2018-12-20

**Authors:** Srinivasarao Thota, Vivek Kumar Srivastav

**Affiliations:** 1 Department of Applied Mathematics, School of Applied Natural Sciences, Adama Science and Technology University, Post Box No. 1888, Adama, Ethiopia; 2 Department of Mathematics, Motihari College of Engineering Motihari, Furshatpur Bariyarpur, Motihari, Bihar 845401 India

**Keywords:** Root of transcendental equations, Regula-Falsi method, Newton–Raphson method, Quadratic convergence, 65Hxx, 65H04

## Abstract

**Objectives:**

The present paper describes a new algorithm to find a root of non-linear transcendental equations. It is found that Regula-Falsi method always gives guaranteed result but slow convergence. However, Newton–Raphson method does not give guaranteed result but faster than Regula-Falsi method. Therefore, the present paper used these two ideas and developed a new algorithm which has better convergence than Regula-Falsi and guaranteed result. One of the major issue in Newton–Raphson method is, it fails when first derivative is zero or approximately zero.

**Results:**

The proposed method implemented the failure condition of Newton–Raphson method with better convergence. Error calculation has been discussed for certain real life examples using Bisection, Regula-Falsi, Newton–Raphson method and new proposed method. The computed results show that the new proposed quadratically convergent method provides better convergence than other methods.

**Electronic supplementary material:**

The online version of this article (10.1186/s13104-018-4008-z) contains supplementary material, which is available to authorized users.

## Introduction

Most of the real life-problems are non-linear in nature therefore it is a challenging task for the mathematician and engineer to find the exact solution of such problems [1, 2]. In this reference, a number of methods have been proposed/implemented in the last two decades [1, 3–8]. Analytical solutions of such non-linear equations are very difficult, therefore only numerical method based iterative techniques are the way to find approximate solution. In the literature, there are some numerical methods such as *Bisection*, *Secant*, *Regula-Falsi*, *Newton–Raphson*, *Mullers* methods, etc., to calculate an approximate root of the non-linear transcendental equations. It is well known [1, 3–11, 14] that all the iterative methods require one or more initial guesses for the initial approximations.

In Regula-Falsi method, two initial guesses are taken in such a way that the corresponding function values have opposite signs. Then these two points are connected through the straight line and next approximation is the point where this line intersect the *x*-axis. This method gives guaranteed result but slow convergence therefore several researchers have improved this standard Regula-Falsi method into different hybrid models to speed up the convergence [1, 3–5, 7, 10, 11, 15, 16]. Thus previously published works have revised/implemented Regula-Falsi method in several ways to obtain better convergence. However, it is found that modified form of Regual-Falsi method becomes more complicated from computational point of view. Therefore, in the present work Regual-Falsi method has been used as its standard form with Newton–Raphson method and found better convergence. Newton–Raphson method is generally used to improve the result obtained by one of the above methods. This method uses the concept of *tangent* at the initial approximation point. The next approximate root is taken those value where the tangent intersect the *x*-axis. So this method fails where tangent is parallel to *x*-axis, i.e. the derivative of the function is zero or approximately zero. The order of convergence of Newton–Raphson method is two, therefore it converges very rapidly than other methods (Bisection, Regula-Falsi, etc.). However it does not always give guaranteed root. Many scientists and engineers have been proposed different hybrid models on Newton–Raphson method [8, 9, 12–14, 17–22].

It is clear from the survey [1–22], that the most of new algorithms are either based on three classical methods namely Bisection, Regula-Falsi and Newton–Raphson or created by hybrid processes. In the present work, the proposed new algorithm is based on standard Regula-Falsi and Newton–Raphson methods, which provides guaranteed results and higher order convergence over Regula-Falsi method. The new proposed algorithm will work even the first derivative equals to zero where Newton–Raphson method fails.

## Main text

Consider a continuous function *f*(*x*) between *a* and *b* such that *f*(*a*) and *f*(*b*) having opposite signs, consequently $f(a) \cdot f(b) < 0$. Without loss of generality, assume that *f*(*a*) is negative and *f*(*b*) is positive, and $|f(a)| < |f(b)|$, hence at least one root lies between *a* and *b*. From Regula-Falsi method, the first approximate root can be calculated by using the formula

1 $$\begin{aligned} x = \frac{af(b)-bf(a)}{f(b)-f(a)}, \end{aligned}$$

and, the first approximate root by using Newton–Raphson method is

2 $$\begin{aligned} x = a - \frac{f(a)}{f'(a)}, \end{aligned}$$

where $f'(a)$ indicates the derivative of *f*(*x*) at $x=a$.

Now, in the present proposed algorithm, we take the average of the iterations in Eqs. (1) and (2) as our first approximate root ${\widehat{x}}$ and follow the conditions given below for further iterations:

- Choose two values *a* and *b* where the root exists as in Regula-Falsi method.
- Select the value such that the corresponding function is closer to zero as *a* and the other one as *b*, i.e. $|f(a)| < |f(b)|$.
- If first derivative at *a* is zero (i.e., $f'(a) =0$) then interchange the values of *a* and *b* i.e., interchange (*a*, *b*) to (*b*, *a*).

The generalization of this process is described in the following section.

### Formulation of proposed algorithm

Recall the Eqs. (1) and (2) in terms of iteration formulae by replacing *a*, *b*, *x* by $x_{n-1}, x_{n+1}, x_n$ respectively, as follows

3 $$\begin{aligned} x_n = \frac{x_{n-1}f(x_{n+1})-x_{n+1}f(x_{n-1})}{f(x_{n+1})-f(x_{n+1})} \end{aligned}$$

and

4 $$\begin{aligned} x_n = x_{n-1} - \frac{f(x_{n-1})}{f'(x_{n-1})}, \end{aligned}$$

where *n* is the iteration number and $|f(x_{n-1})| < |f(x_{n+1})|$. Now the average of Eqs. (3) and (4) is

5 $$\begin{aligned} x_n=\frac{1}{2} \left[ \left( \frac{x_{n-1}f(x_{n+1}) - x_{n+1}f(x_{n-1})}{f(x_{n+1}) - f(x_{n-1})} \right) + \left( x_{n-1} - \frac{f(x_{n-1})}{f'(x_{n-1})} \right) \right] \end{aligned}$$

After simplification of (5), we get

6 $$\begin{aligned} x_n = x_{n-1}-\frac{f(x_{n-1})}{2f'(x_{n-1})}\left( \frac{f(x_{n-1}) - f(x_{n+1})+(x_{n-1}-x_{n+1})(f'(x_{n-1}))}{f(x_{n-1})-f(x_{n+1})} \right) . \end{aligned}$$

The value $x_n$ in Eq. (6) gives the iterative formula with $|f(x_{n-1})| < |f(x_{n+1})|$. If $f'(x_{n-1}) = 0$, then Eq. (6) gives undefined value, then we have to interchange the values $x_{n-1}$ and $x_{n+1}$.

The following Theorem gives the generalization of above formulation.

#### **Lemma 1**

*Let* *f*(*x*)* be a continuous function and* (*a*, *b*)* be a sufficiently small interval such that* $f(a)f(b) < 0$*, and* $f'(x)$* exists on* [*a*, *b*].* Then the approximation of a root of* *f*(*x*)* can be find using the iterative formula given in Eq.* (6).

### Steps for calculating a root

I. Select two initial approximations $x_{n-1}$ and $x_{n+1}$ such that product of the corresponding function values must be negative, i.e. $f(x_{n-1})f(x_{n+1})<0$.
II. Now calculate $x_n$ using the formula given in Eq. (6). Check $f(x_n) = 0$, if so, then $x_n$ is required root and process stop. Otherwise we check the following possible conditions.
  (i) For $f(x_n)f(x_{n-1})<0$, suppose $|f(x_{n-1})| < |f(x_{n})|$ then $x_n$ replace by $x_{n-1}$ and $x_{n-1}$ replace by $x_{n}$.
  (ii) For $f(x_n)f(x_{n+1})<0$, suppose $|f(x_{n})| < |f(x_{n+1})|$ then $x_{n+1}$ replace by $x_{n}$.
III. If $f'(x_{n-1}) \approx 0$ then interchange $x_{n-1}$ and $x_{n+1}$.
IV. Repeat steps I, II and III until we get required approximate solution.

The implementation of the proposed algorithm in Matlab is also discussed (See, Additional file 1). This algorithm would help to implement the manual calculations in commercial packages such as Maple, Mathematica, SCILab, Singular,etc.

### Order of convergence

The order of converges of any iterative method is defined as

7 $$\begin{aligned} |e_{n+1}| \le c |e_n|^p, \end{aligned}$$

where *p* is the order of convergence and *c* is a positive finite constant. The following Theorem shows the order of convergence of the proposed algorithm is quadratic.

#### **Theorem 2**

*Let* $\beta$* be a exact root of a continuous function* *f*(*x*)* and* [*a*, *b*]* be a sufficiently small neighbourhood of* $\beta$.* Then the sequence* $\{x_n\}$* generated by the iterative formula* (6)* is at least quadratically convergent*.

#### *Proof*

If $x_n$ is an approximate value of $\beta$ and $e_n$ is the error of the $x_n$ then

$$\begin{aligned} x_n = \beta + e_n. \end{aligned}$$

In the similar way, $x_{n+1} = \beta + e_{n+1}$ and $x_{n+2} = \beta + e_{n+2}$. By proposed algorithm,

$$\begin{aligned} x_{n+2} = x_{n}-\frac{f(x_{n})}{2f'(x_{n})}\left( \frac{f(x_{n}) - f(x_{n+1})+(x_{n}-x_{n+1})(f'(x_{n}))}{f(x_{n})-f(x_{n+1})} \right) , \end{aligned}$$

putting values of $x_{n},x_{n+1}$ and $x_{n+2}$ in above equation, we get

$$\begin{aligned} \beta + e_{n+2} = \beta + e_{n}-\frac{f(\beta + e_{n})}{2f'(\beta + e_{n})}\left( \frac{f(\beta + e_{n}) - f(\beta + e_{n+1})+(\beta + e_{n}- \beta - e_{n+1})(f'(\beta + e_{n}))}{f(\beta + e_{n})-f(\beta + e_{n+1})} \right) . \end{aligned}$$

After simplification of above equation using Taylor’s series, we get

$$\begin{aligned} e_{n+2} = \left[ e_n e_{n+1} \frac{f''(\beta )}{2f'(\beta )} + e_n^2 \frac{f''(\beta )}{2f'(\beta )} \right] = \frac{f''(\beta )}{2f'(\beta )} \left[ e_n e_{n+1} + e_n^2 \right] , \end{aligned}$$

Putting $\frac{f''(\beta )}{2f'(\beta )} = A$ (constant), then

8 $$\begin{aligned} e_{n+2} = A \left[ e_n e_{n+1} + e_n^2 \right] = A e_n e_{n+1} + A e_n^2. \end{aligned}$$

We have, $|e_{n+2}| = c |e_{n+1}|^p$, $c>0$; $|e_{n+1}| = c |e_{n}|^p$; and $|e_{n}| = c^{-1/p} |e_{n+1}|^{1/p}$. Substituting in Eq. (8) and after simplification, we get

$$\begin{aligned} c_1 e_{n+1}^p &= c_2e_{n+1}^{1+1/p}+c_3e_n^{2} \\ &= c_2'e_{n}^{p+1}+c_3e_n^{2}, \quad \text {where}~~c_2'=c_1c \\ |e_{n+1}|^p &\le c_1^*|e_n|^{p+1}+c_2^*|e_n|^2, \quad \text {where}~~c_1^*=c_2'c_1^{-1}, c_2^*=c_3c_1^{-1} \end{aligned}$$

If $$p=1$$, then

$$\begin{aligned} |e_{n+1}| \le c^*|e_n|^2, \end{aligned}$$

where $c^*=c_1^*+c_2^*$. From Eq. (7), it shows that the iterative formula (6) has quadratic convergent. $\square$

## Results

This section provides three examples to discuss the algorithm presented in “Main text” section  and comparisons are taken into account to conform that the algorithm is more efficient than other existing methods. Moreover, it is also observed that the proposed method takes less time in comparison of Regula Falsi method but takes more convergence time in comparison of Newton–Raphson method.

### *Example 3*

Consider a transcendental equation of the form

9 $$\begin{aligned} xe^x=\cos (x). \end{aligned}$$

We compute a root of Eq. (9) in the interval (0,1) using Bisection, Regula-Falsi, Newton–Raphson and proposed algorithm.

Table 1 shows that the comparison between Bisection, Regula-Falsi, Newton–Raphson and proposed method. The errors given in table are indicating the difference between two consecutive iterations. It is clear that the proposed method rapid convergence towards exact root than bisection and Regula-fasi method. It is not as speed convergent as Newton–Raphson method, but provide guaranteed result.

**Table 1:** Comparison between different methods with errors for Example 3

Ite no.	BM approx. root	% deviation	R-F approx. root	% deviation	N–R approx. root	% deviation	PM approx. root	% deviation
1	0.5000	–	0.3147	–	1.0000	–	0.6573	–
2	0.7500	100	0.4467	100	0.6531	100	0.4886	100
3	0.6250	33.33	0.4940	29.56	0.5313	53.12	0.5165	34.52
4	0.5625	20.00	0.5099	09.57	0.5179	22.91	0.5176	05.40
5	0.5313	11.11	0.5152	03.12	0.5178	02.59	0.5177	00.23
6	0.5156	05.88	0.5169	01.02	0.5178	00.03	0.5177	00.01
7	0.5234	03.03	0.5177	00.10	*0.5178*	*00.00*	*0.5178*	*00.00*
$\vdots$	$\vdots$	$\vdots$	$\vdots$	$\vdots$				
14	$\vdots$	$\vdots$	*0.5178*	*00.00*				
$\vdots$	$\vdots$	$\vdots$						
22	*0.5178*	*00.00*						

Italic values indicate the approximate root at 00.00 % of the deviation using the various methods

It is found that bisection and Regula-Falsi methods converged after 22 and 14 iterations respectively (Table 1), while the proposed algorithm converged in 7th iteration. Thus proposed method is efficient over bisection and Regula-Falsi methods. It is also clear that both of the methods (proposed and Newton–Raphson) are converged in 7th iteration. But one of the main advantage of proposed method is that it gives guaranteed result over the Newton–Raphson method. Therefore, the proposed method is not only reduce the computational affords but also provide the guaranteed result for solving the real life problem.

The error estimation after the 3rd iteration (Table 1), show that the proposed method having $5.4\%$ error in comparison to Bisection ($20\%$), Regula-Falsi ($9.57\%$) and Newton–Raphson ($22.91\%$) methods. Thus, the proposed method is also efficient for error estimation.

Most of the real life problems take too much computational time for convergence because of the complex flow physics and higher degree polynomial equations. Therefore, the proposed method is useful also for solving such the real life problem.

### *Example 4*

Consider a transcendental equation of the form

10 $$\begin{aligned} x\log _{10}(x)-1.2=0. \end{aligned}$$

We compute a root of above equation in the interval (1,3) using Bisection, Regula-Falsi, Newton–Raphson and proposed algorithm.

Table 2 shows that bisection method converged in 21st iteration however the remaining three methods (Regula-Falsi, Newton–Raphson, proposed) converged after 3rd iteration. Thus, as similar to the Example 3, proposed method is efficient to solve this logarithmic problem.

**Table 2:** Comparison between different methods with errors for Example 4

Ite no.	BM approx. root	% deviation	R-F approx. root	% deviation	N–R approx. root	% deviation	PM approx. root	% deviation
1	2.0000	–	2.6767	–	3.7631	–	3.2199	–
2	2.5000	50.00	2.7392	62.64	2.8067	73.43	2.6935	68.94
3	2.7500	20.00	2.7406	02.28	2.7410	34.08	2.7398	19.54
4	2.6250	09.09	2.7406	00.05	2.7406	02.39	2.7406	01.69
5	2.6875	04.76	2.7406	0.001	2.7406	00.01	2.7406	00.03
6	2.7188	02.33	*2.7406*	*00.00*	*2.7406*	*00.00*	*2.7406*	*00.00*
$\vdots$	$\vdots$	$\vdots$						
21	*2.7406*	*00.00*						

Italic values indicate the approximate root at 00.00% of the deviation using the various methods

### *Example 5*

Consider the real root of $f(x) = 1-x^2$ in the interval (0, 2).

In this example, Newton–Raphson method unable to find the real root because of $f'(x)$ is zero at initial approximation $x=0$ as in Eq. (2). However, the proposed method give approximate root with any range of initial approximation as given in Table 3. Thus, the proposed method is also applicable to such equation where $f'(x)$ is zero with any one of the initial approximations. Since $f'(x)=0$ at $a=0$, therefore according to the proposed method, the initial approximations automatically interchange i.e., $a=2$ and $b=0$ as shown in Table 3.

**Table 3:** Numerical results for Example 5

Iter no.	*a*	Approx. root	*b*	% error
1	2.0000	0.8750	0.0000	–
2	0.8750	0.9827	2.0000	100.0
3	0.9827	0.9972	2.0000	10.96
4	0.9972	0.9995	2.0000	01.45
5	0.9995	0.9999	2.0000	00.24
6	0.9999	1.0000	2.0000	00.04
7	1.0000	1.0000	2.0000	00.01
8	1.0000	1.0000	2.0000	00.00

## Limitations

The order of the presented method is quadratic. Even though there are methods with a higher order of convergence, the proposed method is simple and efficient quadratic convergence method.

## Additional file

**Additional file 1.** Implementation of the proposed method in Matlab. In the Additional file, we provide the implementation of the proposed method inMatlab code similar to Regula-Falsi method in [23] by creating a data type NewAlgorithm (f, a, b, esp, n),as given in Additional file, where f is a given transcendental equation, a, b are the initial approximationof the root, esp is the relative error and n is the number of iterations required.

## Abbreviations

BM: Bisection method
R-F: Regula-Falsi
N–R: Newton–Raphson
PM: proposed method
