# Supplementary material for: Quadratically convergent algorithm for computing real root of non-linear transcendental equations
Source: BMC Res Notes. 2018 Dec 20;11:909. doi: 10.1186/s13104-018-4008-z (PMC6302413; doi:10.1186/s13104-018-4008-z)
Supplement: Supplementary file 1 — Additional file 1. Implementation of the proposed method in Matlab. In the Additional file, we provide the implementation of the proposed method inMatlab code similar to Regula-Falsi method in [23] by creating a data type NewAlgorithm (f, a, b, esp, n),as given in Additional file, where f is a given transcendental equation, a, b are the initial approximationof the root, esp is the relative error and n is the number of iterations required. [file 13104_2018_4008_MOESM1_ESM.pdf]

Implementation of the Proposed Method in Matlab

Srinivasarao Thota* and Vivek Kumar Srivastav†

*Department of Applied Mathematics
School of Applied Natural Sciences
Adama Science and Technology University
Post Box No. 1888, Adama, Ethiopia
Email: srinithota@ymail.com and
srinivasarao.thota@astu.edu.et

†Department of Mathematics
Motihari College of Engineering Motihari
Motihari, Furshatpur Bariyarpur, Bihar, India–845401
Email: vivekapril@gmail.com

Implementation

In this file, we provide the implementation of the proposed method (presented in the main paper) in Matlab code by creating a data type `NewAlgorithm(f,a,b,esp,n)`, as given below, where `f` is a given transcendental equation, `a,b` are the initial approximation of the root, `esp` is the relative error and `n` is the number of iterations required.

```
function root = NewAlgorithm(f,a,b,esp,n)
    if f(a)*f(b) > 0
      error('no root')
    end
    if nargin < 5, n = 100; end
    if nargin < 4, esp = 0.000001; end
    iter = 0;
    c = a;
    ea = 0;
    fd = inline(char(diff(formula(f))),'x');
    disp(' --------------------------------------------------------');
    disp(' No    a    Root    b    error    % error');
    disp(' --------------------------------------------------------');
    while (1)
       cold = c;
       if fd(a) == 0
        temp = a; a = b; b = temp;
```

```
   end
   c =((a*f(b)-b*f(a))/(2*(f(b)-f(a))))+((a-(f(a)/fd(a)))/2);
   cnew = c;
   disp(sprintf('%4d %10.4f %10.4f %10.4f %10.2e %8.2f',
         iter+1, a, c, b, abs(cold - cnew), ea));
   iter = iter + 1;
   if c  = 0, ea = abs((c - cold)/c) * 100; end
   check = f(a)*f(c);
   if check < 0
      if abs(f(a)) < abs(f(c))
          c = c;
      else
          b = a;
          a = c;
      end
   elseif check > 0
      if abs(f(c)) < abs(f(b))
          a = c;
      else
          a = b;
          b = c;
      end
   else
      ea = 0;
   end
   if ea <= esp | iter >= n, break, end
end
disp(' --------------------------------------------------');
disp(['Given function f(x) = ' char(f)]);
disp(sprintf('Approximate root = %10.10f',c));
```
